# Hypolipidaemic and Insulin Secretagogue Activities of (R)-(−)-Carvone

**DOI:** 10.21315/mjms2020.27.6.5

**Published:** 2020-12-29

**Authors:** Manal Ahmad Abbas, Ghaleb Ali Oriquat, Manal Mohamed Abbas, Belal Omar Al-Najjar, Yasser Ibrahim Kandil

**Affiliations:** 1 Department of Medical Laboratory Sciences, Faculty of Allied Medical Sciences, Al-Ahliyya Amman University, Amman, Jordan; 2 Department of Biopharmaceutics and Clinical Pharmacy, Faculty of Pharmacy, Al-Ahliyya Amman University, Amman, Jordan; 3 Faculty of Pharmacy, Al-Azhar University, Cairo, Egypt; 4 Department of Pharmaceutical Sciences, Faculty of Pharmacy, Al-Ahliyya Amman University, Amman, Jordan

**Keywords:** 1.1E7 cell line, hyperlipidaemia, insulin, molecular docking, (R)-(−)-carvone, tyloxapol

## Abstract

**Background:**

Dyslipidaemias are common in patients with diabetes mellitus. A high prevalence of type 2 diabetes in hyperlipidaemic patients also exists. The aim of this study was to find a treatment that lowers both blood glucose and lipid levels simultaneously.

**Methods:**

The hypolipidaemic effect of (R)-(−)-carvone was investigated in a tyloxapol-induced hyperlipidaemia mice model. Furthermore, its effect on insulin secretion and proliferation of 1.1E7 human pancreatic β-cells was studied. In addition, using molecular docking, the binding affinity of (R)-(−)-carvone against 3-hydroxy-3-methyl-glutaryl-coenzyme A (HMG-CoA) reductase was estimated.

**Results:**

(R)-(−)-carvone (100 mg/kg) decreased plasma triglyceride, total cholesterol, low-density lipoprotein cholesterol (LDL-C) levels and atherogenic index by 90.6%, 49.3%, 56.6% and 70.3%, respectively, but it had no effect on high-density lipoprotein cholesterol (HDL-C). Furthermore, it increased hepatic triglyceride level and catalase activity by 79.6% and 59.6%, respectively. In-vitro, 500 μM (R)-(−)-carvone increased insulin secretion by 454.4% and proliferation of 1.1E7 cells with no cytotoxic effects up to a concentration of 100 μM. Molecular docking simulation demonstrated a good binding affinity with −5.03 Kcal/mol of (R)-(−)-carvone to HMG-CoA reductase.

**Conclusion:**

The hypolipidaemic effect of (R)-(−)-carvone is comparable to that of fenofibrate. (R)-(−)-carvone has the advantage over fenofibrate of not producing hypoglycaemia in animals. Furthermore, (R)-(−)-carvone increased proliferation and insulin secretion of human pancreatic β-cells.

## Introduction

Dyslipidaemias are common in patients with diabetes mellitus. A high prevalence of type 2 diabetes in hyperlipidaemic patients also exists (1). Epidemiologic studies have demonstrated a strong correlation between serum lipids and the risk of atherosclerosis. Therefore, multifactorial intervention strategies are used to control lipid and blood glucose levels simultaneously to achieve maximal reductions in cardiovascular risk (2).

Current anti-hyperlipidaemic drugs include statins and fibrates. Statins inhibit the biosynthesis of cholesterol and fibrates enhance the clearance of triglycerides (TGs) (3). The side effects of statins and fibrates are well documented (4, 5). For this reason, many studies have been conducted to evaluate the potential lipid-lowering activity of synthetic and natural compounds (6). It has been suggested that herbal remedies are among the best existing alternative therapies and have been used since ancient times (7) and they have contributed to the development of important drugs used currently in modern medicine (8).

Carvone is a volatile monocyclic terpenoid found in many essential oils. It has two enantiomers with different biological activities. The (S)-(+)-carvone is found in caraway seeds and has a caraway odour, whereas the (R)-(−)-carvone is found in mint leaves and has a mint odour (9). Carvone has several applications – it is used in fragrances and flavours and also as a potato sprouting inhibitor and antimicrobial agent (9).

In L-NAME (Nω-nitro-L-arginine methyl ester hydrochloride) hypertensive rats, (S)-(+)-carvone supplementation prevented the development of hyperlipidaemia (10). Furthermore, Mentha spicata containing 51.7% (R)-carvone had a hypocholesterolaemic effect in diabetic rats (11). The hypolipidaemic activity of the two enantiomers of carvone may differ since some enzymes involved in lipid metabolism are stereospecific, such as the enzyme 3-hydroxy-3-methylglutaryl–coenzyme A (HMG-CoA) reductase, which catalyses the rate-limiting step in cholesterol biosynthesis (12). To the best of our knowledge, no previous study has investigated the effect of (R)-(−)-carvone in terms of its ability to lower blood cholesterol and TG levels.

The limited availability of human islets of Langerhans for research has prompted researchers to study the physiological regulation of insulin secretion in non-human models (13). However, interspecies differences do exist in terms of insulin secretion. For example, human β-cells differ from rodent cells in several aspects of voltage-gated ion channels. Moreover, human β-cells have a lower set point for glucose-stimulated insulin secretion compared with rodent β-cells (13). In a streptozotocin (STZ)-induced diabetic rat model, (R)-(−)-carvone reduced plasma glucose, improved insulin levels and modulated key hepatic enzymes of glucose metabolism (14). However, studying insulin secretion in rodents does not necessarily equate to studying insulin secretion in humans. Therefore, this study was designed to test the effect of (R)-(−)-carvone on insulin secretion and proliferation of human β-cells and to investigate the hypolipidaemic activity of (R)-(−)-carvone using a Triton WR 1339 (Tyloxapol)-induced hyperlipidaemic mouse model.

## Methods

### Chemicals, Kits and Reagents

(R)-(−)-carvone and tyloxapol (Triton WR 1339) were purchased from Sigma-Aldrich (USA) and fenofibrate (Trigless) was purchased from Jordan Sweden Medical and Sterilization Company (JOSWE), Jordan. Kits for glucose (code: 11503), plasma TGs (code: 11529), total cholesterol (TC) (code: 11505), high-density lipoprotein cholesterol (HDL-C) (code: 11648) and low-density lipoprotein cholesterol (LDL-C), and precipitating reagent (code: 11579) were purchased from BioSystems (S.A., Barcelona Spain). Total glutathione (GSH) (catalog number: 703002) and catalase assay (catalog number: 707002) were supplied by Cayman Chemical Co., USA. The insulin-secreting human pancreatic β-cell line 1.1E7 was obtained from the European Collection of Cell Cultures (ECACC), United Kingdom. The MTT kit was from Promega (USA). All other chemicals were of analytical grade. (R)-(−)-carvone was emulsified in 2% Tween 20 for in vivo studies. For in vitro studies, (R)-(−)-carvone was dissolved in dimethyl sulfoxide (DMSO) and diluted to prepare the various concentrations so that the final DMSO concentration was less than 0.5%. Fenofibrate and tyloxapol were dissolved in normal saline. All reagents were freshly prepared before use.

### Hypolipidaemic Effect of (R)-(−)-Carvone in Tyloxapol-Induced Hyperlipidaemia

The study was conducted in the experimental animal laboratory of the Faculty of Pharmacy, Al-Ahliyya Amman University, Jordan. Animal use and care were in accordance with the Helsinki Declaration and ethically approved by the Ethical Committee for research on animals at Al-Ahliyya Amman University (ethical approval number AAU-3/5/2014-2015).

**Figure 1:**
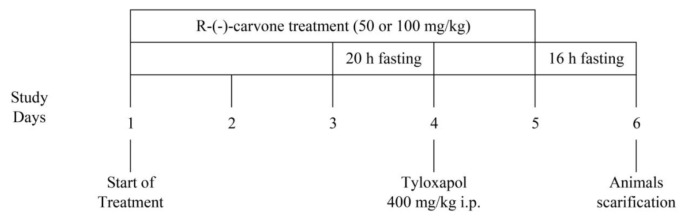
Schematic representation of the experimental model

Male BALB/c mice weighing 22 g–27 g were kept under standard laboratory conditions with a pellet diet and water was made available ad libitum. Mice were housed, fed and treated in accordance with the in-house ethical guidelines for animal protection. They were divided into five groups (eight mice per group) and administered various intraperitoneal (IP) treatments. Groups I and II were administered a vehicle (2% tween 20). Group III was administered the standard drug (100 mg/kg fenofibrate). Lastly, Groups IV and V were administered 50 mg/kg of (R)-(−)-carvone or 100 mg/kg (R)-(−)-carvone. The choice of (R)-(−)-carvone doses was based on previously conducted pilot studies, whereas the choice of fenofibrate dose was according to Lambert et al. (15). On the third day, the animals were fasted for 20 h (with only access to water), and Tyloxapol was dissolved in normal saline and was intraperitoneally injected on day 4 at a dose of 400 mg/kg of body weight to all groups except Group I (Figure 1). The choice of tyloxapol dose was according to Ferreira et al. (16). After 24 h from the last dose of vehicle, (R)-(−)-carvone or fenofibrate, the animals were fasted for 16 h. One millilitre of blood was withdrawn using retro-orbital plexus and collected in EDTA vacutainer tubes and was centrifuged at 1,000 g for 10 min. The plasma was separated and kept at −20 °C for the determination of biochemical parameters. Fasting blood sugar (FBS), TGs, TC, HDL-C and LDL-C were performed following the manufacturer’s instructions. Atherogenic index (AI) was calculated as AI = TC – HDL-C/HDL-C for each sample (17).

After cervical dislocation, liver specimens were removed and cut into two halves. One piece was stored in 10% buffered formalin for further histopathological studies, and the remainder was kept at −20 °C for further antioxidant tests and for the determination of TG content in the liver. Five micrometre paraffin-embedded sections were prepared and examined under a light microscope.

### Measurement of TG Content in Liver

Liver TG content was determined by extraction of frozen liver tissue with isopropanol. Briefly, 50 mg of the liver was homogenised in 1 mL of isopropanol, centrifuged at 10,000 g for 15 min. Tissue TG level was assayed in the supernatant using a Biosystem kit and the results were expressed as mg of TGs per gram of tissue weight.

### Liver Homogenisation for Oxidative Stress Parameters

Liver samples (100 mg) were homogenised in 1 mL of ice-cold cell lysing buffer (catalog number: 895347, R&D Systems Inc. Minneapolis, USA) using Teflon homogeniser (Potter-Elvehjem). The homogenate was then cold-centrifuged at 10,000 g for 10 min and the supernatant was used for total protein assay according to the method in Lowry et al. (18). Part of the supernatant was deproteinised using 5% metaphosphoric acid solution and used for measurement of total GSH level; another part was used for determination of catalase activity.

Total GSH level was measured according to the Eyer method (19). Simply, the method depends on the reduction of Ellman’s reagent (5,5′-dithiobis-2-nitrobenzoic acid) by sulfhydryl (SH) group to produce a yellow colour compound with a maximum absorbance at 405 nm–412 nm. The GSH concentration was expressed as nM/g protein. Catalase activity was assayed in liver homogenate according to manufacturer guidelines, depending on both catalytic and peroxidic activities of catalase and expressed in mg protein as a number of H_2_O_2_ nmol was decomposed per min (20).

### Molecular Docking

Molecular docking simulation of (R)-(−)-carvone was conducted to study the intermolecular interactions against HMG-CoA reductase enzyme utilising AutoDockTools 4.2 (21). Approximately 42 Protein Data Bank (PDB) crystal structures were found in the PDB, with only 10 of them having a resolution > 2.0. The crystal structure with a PDB code of 2R4F was selected for the current study (22). Moreover, molecular docking simulation was performed on the co-crystallised inhibitor (structure code: RIE) in order to validate the docking parameters (23). Both compounds were uploaded in AutoDockTools and the Gasteiger charges were added to each compound (24). These files were converted to the AutoDockTools pdbq format, indicating all possible rotatable bonds in the structure. Similarly, the protein crystal structure was uploaded in AutoDockTools, with only chains A and B being selected and adding polar hydrogens, Kollman charges and solvation parameters (24). The grid box size was set to 22.5 Å for all axes (x, y and z) to cover the binding site of the protein. With 100 runs for each compound, docking simulations were conducted using the Lamarckian genetic algorithm (LGA) (25) parameters. After completing the docking simulation, AutoDockTools produced the output information of the docked coordinates and the free energy of binding in the docking log file (dlg).

### Effect of (R)-(−)-Carvone on Human Pancreatic β-Cell Line (1.1E7) Viability In Vitro

Cell viability was assessed by an MTT kit [3-(4,5-dimethylthiazolyl)-2,5-diphenyl-tetrazolium bromide]. Cells were subcultured in RPMI-1640 with L-glutamin complete media in 96-well plates (20,000 cells/well) and left in a CO_2_ incubator at 37 ºC overnight. Then, different concentrations of (R)-(−)-carvone (0.78, 1.56, 3.12, 6.24, 12.48, 25, 50, 100, 250, 500 and 1,000 μM, *n* = 3) were added. After 48 h of incubation, the effects of (R)-(−)-carvone on cell viability were evaluated according to manufacturer instructions.

### Effect of (R)-(−)-Carvone on Insulin Release from Human Pancreatic β-Cell Line (1.1E7)

Twenty-four hours prior to performing the insulin release studies, cells were harvested from routine culture seed 500,000 cells per well/mL media into six-well plates. The six-well plates were returned to the incubator for overnight culture. Prior to the acute tests (insulin release studies), media was removed by inverting the plate and incubating cells in each well with 1.5 mL of Krebs-Ringer bicarbonate buffer (KRBB) (115 mM NaCl, 4.7 mM KCl, 1.28 mM CaCl.6H_2_O, 1.2 mM KH_2_PO_4_, 1.2 mM MgSO_4_.7H_2_O) containing 1.1 mM glucose for 40 min at 37 °C. The preincubation reagent was removed. Then, pre-warmed, acute test samples in triplicates were used, made up KRBB for 20 min at 37 °C (1.5 mL/well). After 20 min, plates were taken from the incubator and 1 mL of supernatant was carefully removed from each well and placed into pre-labeled tubes. Samples were stored immediately at −20 °C until further analysis. Insulin level was measured using chemiluminescent microparticle immunoassay technology (ARCHITECT Insulin assay, Abbott).

### Effect of (R)-(−)-Carvone on Proliferation of Human Pancreatic β-Cell Line (1.1E7)

Proliferation of human pancreatic β-cell line (1.1E7) cells cultured on polylysine-coated cover slips (Thermanox, USA) was evaluated by the addition of 5-bromo-2′-deoxy-uridine (BrdU) (Sigma-Aldrich, USA) within the log phase 24 h after preincubation with (R)-(−)-carvone. BrdU was left in the medium for 4 h. Then, cells were washed to remove excess BrdU. The incorporation of BrdU into cellular DNA was detected using immunofluorescence assay for the detection of BrdU (Roche, Germany, catalog number: 11 296 736 001). At the end of experiment, cells were examined using the Zeiss fluorescent microscope (Axioi imagerZ), which was equipped with Metasystems software (Germany).

### Statistical Analysis

All descriptive statistics, analyses and graphics were performed using GraphPad Prism version 6 (GraphPad Software, San Diego, USA). Data were expressed in tables and figures as means and standard deviations (SD) at 95% confidence intervals (CI). All data passed Shapiro-Wilk test normality test. Therefore, one-way analysis of variance (ANOVA) followed by Tukey-Kramer post analysis procedure was used to compare the means of all groups for all studied parameters. Differences between means were considered statistically significant at *P* < 0.05.

## Results

### Hypolipidaemic Effect of (R)-(−)-Carvone

As shown in (Table 1), tyloxapol increased significantly plasma TGs, TC, LDL-C, and AI, whereas it decreased HDL-C. (R)-(−)-carvone (100 mg/kg) and fenofibrate (100 mg/kg) significantly lowered plasma TGs, TC, LDL-C and AI. Fenofibrate but not (R)-(−)-carvone (100 mg/kg) increased HDL-C. Furthermore, fenofibrate significantly lowered FBS. Tyloxapol significantly decreased hepatic TGs, whereas both fenofibrate and (R)-(−)-carvone increased it.

**Figure 2:**
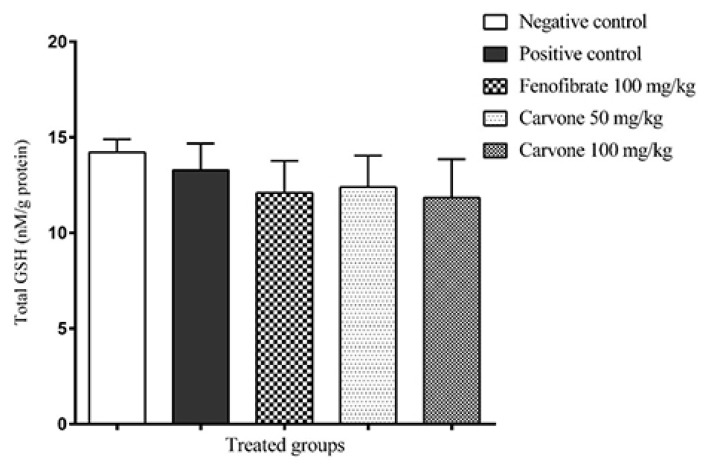
Total glutathione in the liver of control, tyloxapol and fenofibrate 100 mg/kg, and (R)-(−)-carvone doses (50 mg/kg and 100 mg/kg) treated animals (*n* = 8). Values are expressed as means with 95% CI of the mean

No significant difference in hepatic total GSH levels was found among the studied groups (Figure 2). GSH levels were (14.2 ± 0.74) [CI: 13.5–14.9], (13.3 ± 1.68) [CI: 11.9–14.7], (12.1 (1.99) [CI: 10.4–13.8], (12.4 ± 1.55) [CI: 10.8–14.0] and (11.9 ± 2.18) [CI: 9.84–13.9] nM/g protein for the negative control, positive control (tyloxapol-treated), fenofibrate, 50 mg/kg (R)-(−)-carvone and 100 mg/kg (R)-(−)-carvone, respectively.

**Figure 3:**
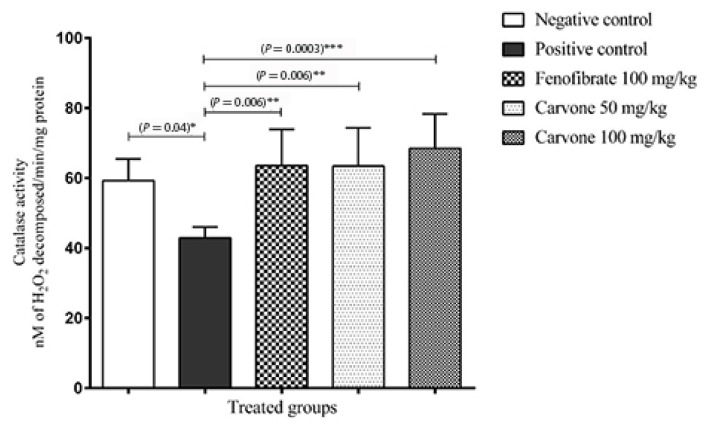
Catalase activity in the liver of control, tyloxapol and fenofibrate 100 mg/kg, and (R)-(−)-carvone doses (50 mg/kg and 100 mg/kg) treated animals (*n* = 8). Values are expressed as means with 95% CI of the mean. *P*-values * < 0.05; ** < 0.01; *** < 0.001

Catalase activity by nM of H_2_O_2_ decomposed/min/mg protein was significantly reduced by tyloxapol injection (42.9 ± 2.90) [CI: 39.9–46.0]) as compared to the negative control group (59.3 ± 6.70) [CI: 53.1–65.5]). This effect was reversed by fenofibrate (63.6 ± 11.2) [CI: 53.3–73.9]) as well as by (R)-(−)-carvone doses of 50 mg/kg (63.4 ± 11.8) [CI: 52.6–74.3]) and of 100 mg/kg (68.5 ± 11.8) [CI: 58.6–78.3]) (Figure 3).

**Figure 4:**
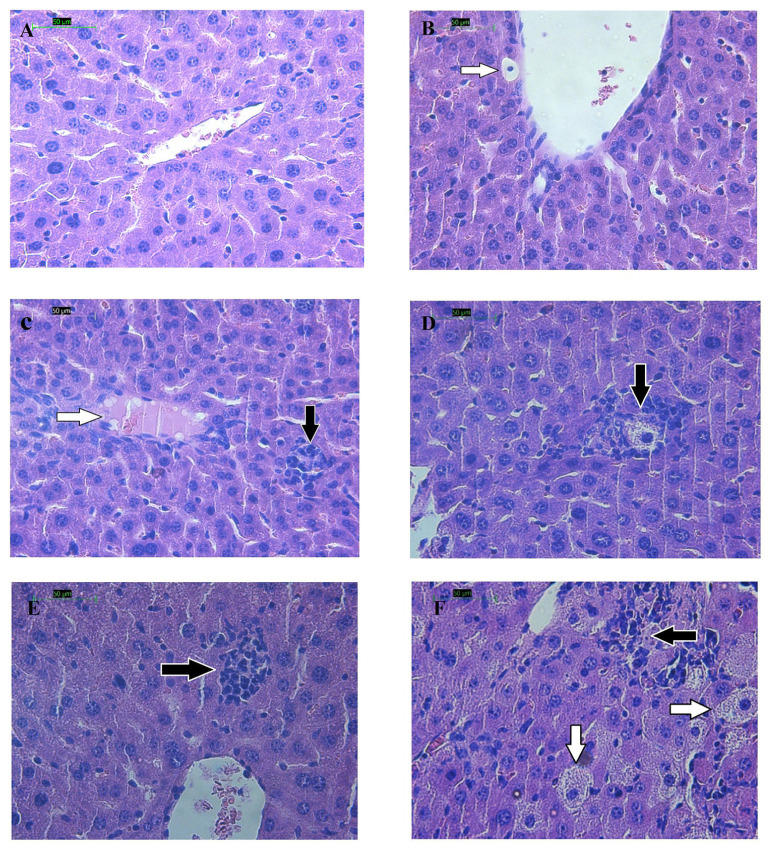
(A) Liver histology; (B, C, D) Normal control: Tyloxapol-treated mice with apoptotic cells seen in (B) and eosinophilic cytoplasm in (C) (white arrow) and infiltration with inflammatory cells (black arrow) in (D); (E) Livers treated with 100 mg/kg (R)-(−)-carvone with inflammatory cell infiltration (black arrow); (F) Fenofibrate-treated group. Note inflammatory cell infiltration (black arrow) and lipid accumulation in hepatocytes (white arrow) (H & E stain)

Histological examination of the liver in the control group revealed normal architecture. The hepatocytes were arranged in plates around the central vein with sinosoids between plates. The liver of the tyloxapol-treated group demonstrated degenerative changes with inflammatory cells (Figures 4B, 4C and 4D). Few hepatocytes were loaded with fat droplets. (R)-(−)-carvone and fenofibrate-treated livers were also infiltrated with inflammatory cells. Signs of apoptosis and fat accumulation inside few hepatocytes were also detected in both groups (Figures 4E and 4F).

### Molecular Docking

**Figure 5:**
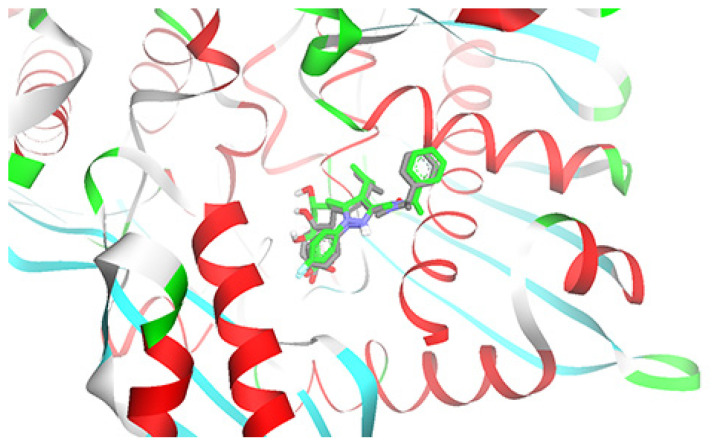
Flat ribbon representation of HMG-CoA reductase (PDB code: 2R4F) crystal structure bound with the co-crystallised ligand (grey) and the re-docked conformation (green)

**Figure 6:**
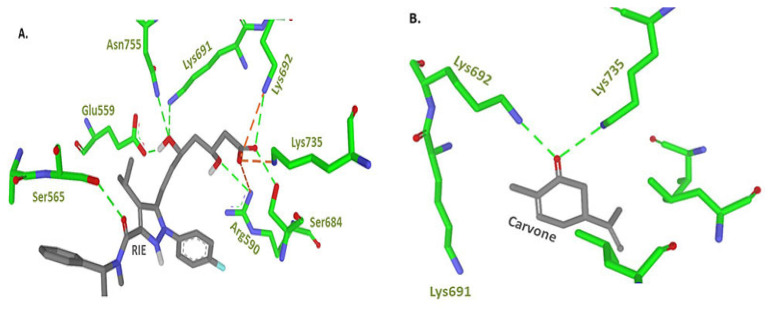
Stick representation of intermolecular interaction between **A**. co-crystallised ligand (RIE) and **B**. (R)-(−)-carvone against HMG-CoA reductase (PDB code: 2R4F). The hydrogen bond interactions are represented by green-dashed lines while the red lines represent ionic interactions. Created by Biovia Discovery Studio visualiser (26)

The co-crystallised ligand (structure code: RIE) was successfully re-docked against 2R4F crystal structure with a Root Mean Square Deviation (RMSD) of 1.2 Å and a free energy of binding of −8.3 Kcal/mol. Generally, molecular docking simulations that produce an RMSD values of less than 2.0 Å are considered to have performed successfully (23), as seen in Figure 5. Similar parameters were used to dock (R)-(−)-carvone in the binding site. The lowest binding energy pose with an R-isomer of carvone demonstrated an energy of −5.03 Kcal/mol, forming a hydrogen bond interaction with Lys692 and Lys735, as well as several hydrophobic interactions with Val683, Leu853 and Leu857, as depicted in Figure 6B. Interestingly, both Lys692 and Lys735 were observed in the intermolecular interaction network between the co-crystallised ligand (structure code: RIE) and the active site, as shown in Figure 6A. Other important hydrogen bond interactions were primarily with Glu559, Ser565, Ser684, Lys691 and Asn755. Moreover, a strong ionic interaction was uncovered with Arg590, Lys692 and Lys735.

### Effect of (R)-(−)-Carvone on 1.1E7 Viability, Insulin Release and Proliferation of 1.1E7

No toxic effect was observed for (R)-(−)-carvone on 1.1E7 cells up to 100 μM after 24 h incubation (previous study done in the same lab). On the other hand, the cytotoxicity for concentrations of 250 μM, 500 μM and 1000 μM were 24.24%, 23.33% and 23.42%, respectively, after 48 h of incubation.

**Figure 7:**
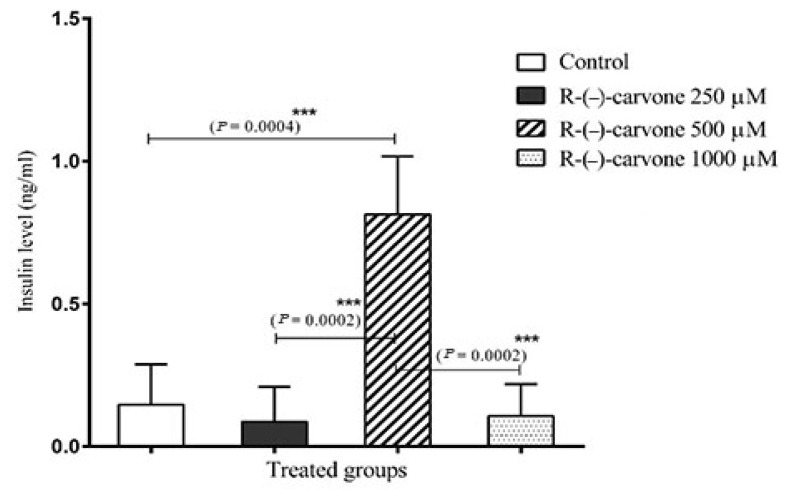
Effect of (R)-(−)-carvone (250 μM, 500 μM and 1000 μM) on insulin secretion in 1.1E7 human β-cell line. Results are expressed as mean with 95% CI of the mean for *n* = 3 replicates

**Figure 8:**
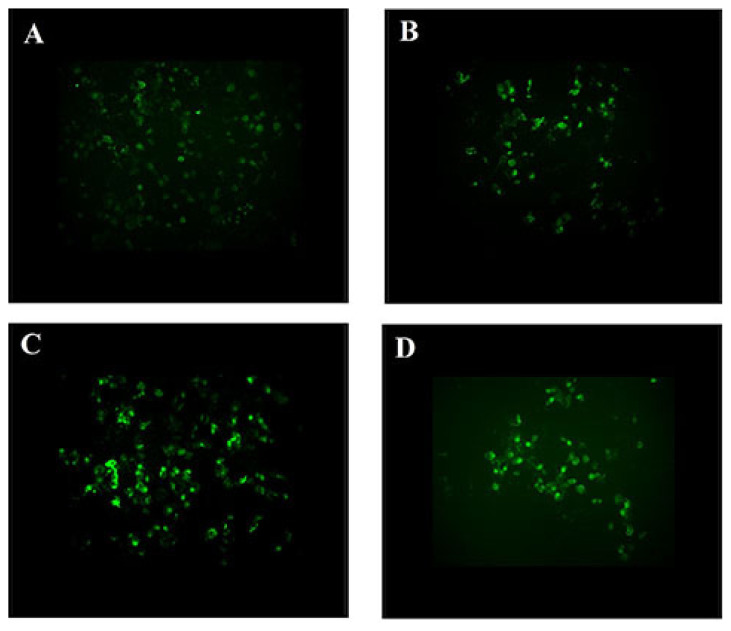
Effect of (R)-(−)-carvone on the proliferation of 1.1E7 human β-cell line in the presence of fluorescent dye. (A) 1.1E7 cells without (R)-(−)-carvone (control); (B) 1.1E7 cells pre-incubated with 1000 μM; (C) 1.1E7 cells pre-incubated with 500 μM; and (D) 1.1E7 cells pre-incubated with 250 μM (R)-(−)-carvone

Five hundred micrometre of (R)-(−)-carvone increased insulin secretion by 454.4% compared to the control in 1.1E7 human β-cell line after 24 h incubation. Other concentrations of (R)-(−)-carvone were ineffective at enhancing insulin secretion (Figure 7). Furthermore, (R)-(−)-carvone increased the proliferation of 1.1E7 human β-cell line as evidenced by the increase in fluorescence due to the fluorescent-labeled BrdU after 24 h incubation (Figure 8).

## Discussion

### Hypolipidaemic Effect of (R)-(−)-Carvone

Hyperlipidaemia is defined as high levels of lipids, cholesterol and TGs, circulating in the bloodstream. Epidemiological studies have demonstrated that hyperlipidaemia is the most prevalent indicator of susceptibility to atherosclerosis and coronary heart diseases (CHD). Therefore, decreasing plasma lipids play a major role in the treatment and prevention of CHD (6). Furthermore, hyperlipidaemia and hypercholesterolemia are not only secondary metabolic dysregulations associated with diabetes but also represent increased risk factors for its development (27).

The tyloxapol-induced hyperlipidaemia model is widely used for screening natural and synthetic hypolipidaemic drugs (28). Tyloxapol (triton WR-1339) blocks clearance of TG-rich lipoproteins to induce acute hyperlipidaemia in different animal species. Additionally, tyloxapol increases hepatic HMG-CoA reductase activity and cholesterol synthesis (29). In this study, tyloxapol administration to mice resulted in a significant increase in plasma TGs, TC, LDL-C and AI levels and lowered HDL-C. Treatment with the same dose of fenofibrate or (R)-(−)-carvone resulted in reduction of plasma TGs, TC, LDL-C, and AI levels. However, fenofibrate but not (R)-(−)-carvone was able to increase HDL-C. The hypolipidaemic effect of fenofibrate in tyloxapol-induced hyperlipidaemic model is similar to its hypolipidaemic effect in humans (30). However, a hypoglycaemic state was produced by fenofibrate but not by (R)-(−)-carvone in mice in the tyloxapol-hypolipidaemic model. This finding suggests an advantage of using (R)-(−)-carvone as a hypolipidaemic agent over fenofibrate since it did not produce hypoglycaemia in normoglycaemic mice.

The hypolipidaemic effect of plants containing carvone was reported. For example, Carum carvi containing (S)-(+)-carvone significantly decreased serum TGs, LDL-C, and total cholesterol levels in rats (31). Similarly, oral administration of Anethum graveolens essential oil containing 28% S-carvone (32) to rats reduced TC, TGs and LDL-C and increased significantly HDL-C. S-carvone also lowered cholesterol level in two rat disease models: in diabetic rats (11) and in L-NAME hypertensive rats (10). It is important to consider that the metabolism of R- and S-carvone is stereoselective in phase-I and phase-II. Furthermore, the enzyme HMG-CoA reductase, which catalyses the rate-limiting step in cholesterol biosynthesis is stereospecific (12). Therefore, the current work represents the first report on the hypolipidaemic effect of (R)-(−)-carvone, the other enantiomer of carvone.

In the present study, fenofibrate increased significantly hepatic TG content in liver specimens of animals treated with tyloxapol compared to untreated mouse liver specimens. The histopathological examination of liver confirmed this finding since fat-filled hepatocytes were present more commonly in fenofibrate-treated rats. It has been reported that intracellular fatty acid flux in the liver is regulated by key proteins such as acyl coenzyme A synthetase, which catalyses the esterification of fatty acids, thus favouring their cellular retention. By increasing the expression of acyl coenzyme A synthetase, peroxisome proliferator-activated receptor-alpha (PPARα) agonists such as fibrates increase fatty acid uptake by the liver (33, 34). This may explain the higher hepatic TG content in the fenofibrate/tyloxapol-treated group. (R)-(−)-carvone also increased TGs in the liver of tyloxapol-treated mice. Future studies may clarify the effect of (R)-(−)-carvone on TGs metabolism and accumulation in liver and its interaction with PPARα receptor.

### Oxidative Stress Parameters

In the present work, R-(−)-carvone had no effect on total GSH level in liver tissue. Surprisingly, it has been reported that D-carvone decreased GSH level in most tissues (35). However, catalase activity in liver tissue was significantly lowered by tyloxapol treatment as in other studies (36). Treatment with fenofibrate or (R)-(−)-carvone effectively increased catalase activity. It has been demonstrated that increased oxidative stress is a deleterious factor leading to insulin resistance and dyslipidaemia (37). Therefore, treatment with antioxidants would be a beneficial strategy in these diseases.

### Molecular Docking

Based on our molecular docking simulation study, it was predicted that (R)-(−)-carvone displayed good binding affinity to HMG-CoA (−5.03 Kcal/mol). Therefore, enzyme inhibitory assay should be conducted to affirm the results of molecular docking. In our in vivo study, (R)-(−)-carvone treatment resulted in reduction of plasma TC. It is not clear whether its mechanism of action is due to the inhibition of HMG-CoA and/or other mechanisms.

### Effect of (R)-(−)-Carvone on 1.1E7 Viability, Insulin Release and Proliferation of 1.1E7

Another activity of (R)-(−)-carvone investigated in this work is its effect on proliferation and insulin secretion of human pancreatic β-cells (1.1E7). In 24 h incubation, (R)-(−)-carvone increased the proliferation of 1.1E7 human β-cell line as evidenced by the increase in fluorescence due to the fluorescent-labelled BrdU (Figure 8). Furthermore, it had no toxic effect up to 100 μM and low toxicity (less than 24%) up to 1,000 μM. It is well established that pancreatic β-cell mass is reduced in both type 1 and type 2 diabetes mellitus (38). Therefore, stimulating the endogenous regeneration of islets will be an essential approach for the treatment of insulin-dependent diabetes mellitus (39). Furthermore, (R)-(−)-carvone increased insulin secretion in human pancreatic cell lines. A similar effect was exerted by (R)-(−)-carvone in STZ-diabetic rats (40). (R)-(−)-carvone is a dietary constituent approved by the FDA (41), and this favours the use of (R)-(−)-carvone in future clinical trials to study its effect in diabetic patients.

## Conclusion

The present study reveals that the hypolipidaemic effect of (R)-(−)-carvone on plasma TGs and cholesterol levels is comparable to that of the standard drug fenofibrate. (R)-(−)-carvone has the advantage over fenofibrate in terms of not inducing hypoglycaemia in normoglycaemic animals. Furthermore, (R)-(−)-carvone increased proliferation and insulin secretion of human pancreatic β-cells in vitro. Future clinical studies are needed to test the effectiveness of (R)-(−)-carvone in treating hyperlipidaemia and to test its effects on insulin release in humans.

## Figures and Tables

**Table 1:** Effects of (R)-(*−*)-carvone and fenofibrate on plasma lipids profile, FBS and hepatic TG content in tyloxapol-induced hyperlipidaemic model

	Negative control	Positive control (vehicle + tyloxapol)	Fenofibrate (100 mg/kg) + tyloxapol	(R)-(−)-carvone-treated groups + tyloxapol	
				50 mg/kg	100 mg/kg
TGs (mg/dL)	77.9 (20.3) [59.1–96.7]	960 (317)^a^ [734–1188]	82.1 (16.1)^b^ [68.6–95.5]	929 (242)^a^^c^ [726–1131]	89.7 (46.4)^b^^d^ [46.8–133]
TC (mg/dL)	101 (14.6) [87.4–114]	290 (117)^a^ [193–388]	104 (26.5)^b^ [81.6–126]	276 (80.6)^a^^c^ [208–343]	147 (29.4)^b^^d^ [123–172]
HDL-C (mg/dL)	62.6 (7.83) [55.3–69.8]	34.2 (10.3)^a^ [25.6–42.9]	59.2 (15.7)^b^ [46.1–72.3]	48.0 (11.2) [38.7–57.4]	46.5 (7.42) [40.3–52.7]
LDL-C (mg/dL)	27.4 (12.7) [15.6–39.2]	182 (73.3)^a^ [121–244]	25.1 (13.4)^b^ [13.9–36.3]	189 (68.3)^a^^c^ [132–246]	78.9 (31.5)^b^^d^ [52.5–105]
AI	0.63 (0.26) [0.39–0.86]	7.38 (1.19)^a^ [6.39–8.38]	0.82 (0.50)^b^ [0.40–1.24]	5.17 (2.73)^a^^b^^c^ [2.88–7.45]	2.19 (0.62)^b^^d^ [1.68–2.71]
FBS (mg/dL)	94.9 (7.61) [87.9–102]	118 (29.0) [93.8–142]	57.7 (13.3)^a^^b^ [46.7–68.8]	108 (17.3)^c^ [93.7–123]	98.6 (31.0)^c^ [72.7–125]
Hepatic TGs (mg/g tissue)	28.3 (6.07) [22.7–33.9]	19.2 (4.81) [15.2–23.2]	43.5 (10.7)^a^^b^ [33.6–53.4]	30.6 (8.52)^b^^c^ [22.8–38.5]	34.5 (3.47)^b^ [31.3–37.7]

Notes: Values are expressed as mean (SD) [95% CI of the mean];

a significantly different from negative control group;

b significantly different from positive control group;

c significantly different from Fenofibrate-treated (100 mg/kg) group;

d significantly different from (R)-(*−*)-carvone-treated (50 mg/kg) group; Number of mice/group = 8; significant difference was considered if *P-*value < 0.05
